# Tumor Suppressor miR-27a-5p and Its Significance for Breast Cancer

**DOI:** 10.3390/biomedicines12112625

**Published:** 2024-11-17

**Authors:** Paola Parrella, Raffaela Barbano, Katharina Jonas, Andrea Fontana, Serena Barile, Michelina Rendina, Antonio lo Mele, Giuseppina Prencipe, Luigi Ciuffreda, Maria Grazia Morritti, Vanna Maria Valori, Paolo Graziano, Evaristo Maiello, Massimiliano Copetti, Martin Pichler, Barbara Pasculli

**Affiliations:** 1 Laboratory of Oncology, Fondazione IRCCS “Casa Sollievo della Sofferenza”, 71013 San Giovanni Rotondo, Italy; pparrella@operapadrepio.it (P.P.); r.barbano@operapadrepio.it (R.B.); serena.barile95@gmail.com (S.B.); m.rendina@operapadrepio.it (M.R.); a.lomele@operapadrepio.it (A.l.M.); g.prencipe@operapadrepio.it (G.P.); 2 Unit of Transfusion Medicine, Chemical-Clinical Analysis Laboratory, Fondazione IRCCS “Casa Sollievo della Sofferenza”, 71013 San Giovanni Rotondo, Italy; 3 Division of Oncology, Department of Internal Medicine, Medical University of Graz, 8036 Graz, Austria; katharina.jonas@medunigraz.at (K.J.); martin.pichler@medunigraz.at (M.P.); 4 Research Unit for Non-Coding RNA and Genome Editing, Medical University of Graz, 8010 Graz, Austria; 5 Unit of Biostatistics, Fondazione IRCCS “Casa Sollievo della Sofferenza”, 71013 San Giovanni Rotondo, Italy; a.fontana@operapadrepio.it (A.F.); m.copetti@operapadrepio.it (M.C.); 6 Dipartimento di Bioscienze, Biotecnologie, e Ambiente, Università di Bari “Aldo Moro”, 70121 Bari, Italy; 7 Breast Unit, Fondazione IRCCS “Casa Sollievo della Sofferenza”, 71013 San Giovanni Rotondo, Italy; l.ciuffreda@operapadrepio.it; 8 Unit of Oncology, Fondazione IRCCS “Casa Sollievo della Sofferenza”, 71013 San Giovanni Rotondo, Italy; m.morritti@operapadrepio.it (M.G.M.); v.valori@operapadrepio.it (V.M.V.); e.maiello@operapadrepio.it (E.M.); 9 Department of Radiological, Oncological and Pathological Sciences, Sapienza University of Rome, 00185 Rome, Italy; paolo.graziano@uniroma1.it; 10 Unit of Pathology, Fondazione IRCCS “Casa Sollievo della Sofferenza”, 71013 San Giovanni Rotondo, Italy; 11 Translational Oncology, II. Med Clinics University of Augsburg, 86159 Augsburg, Germany; 12 Division of Oncology, Hematology and Palliative Care, General Clinics, 7400 Oberwart, Austria

**Keywords:** breast cancer, metastases, microRNA, miR-27a-5p, prognosis

## Abstract

**Background:** MicroRNAs are well established as master regulators of carcinogenesis and potential biomarkers in breast cancer (BC). In a preliminary effort, we found miR-27a-5p to be significantly downregulated in experimentally derived mammospheres and BC patients from The Cancer Genome Atlas Breast Invasive Carcinoma (TCGA-BRCA) dataset. **Objectives.** Herein, we sought to investigate the putative involvement of miR-27a-5p in promoting a migratory phenotype of breast cancer cells, and establish whether miR-27a-5p is associated with patient clinicopathological characteristics. **Methods:** miR-27a-5p capability of inducing a metastasis-prone cell phenotype was analyzed in SUM159 and MDA-MB-231, both representing the triple negative BC subtype. miR-27a-5p expression profile was carried out in a cohort of 232 BC patients and normal breast tissues (NBTs) by RT-qPCR. **Results:** Transient miR-27a-5p inhibition did not affect cell proliferation but led to a significant increase of cell migration in knocked-down compared to control cells. Following quantification in the patient cohort, miR-27a-5p was found higher in NBTs (Median 2.28, IQR 1.50–5.40) and pre-invasive breast lesions (Median 3.32, IQR 1.68–4.32) compared to tumors. In particular, miR-27a-5p was less expressed in patients with synchronous (Median 1.03, IQR 0.83–1.58) or metachronous (Median 1.83, IQR 1.29–3.17) metastases than in patients free from metastases after a 5-year follow-up (Median 2.17, IQR 1.19–3.64), suggesting that miR-27a-5p expression is negatively correlated with breast pathology evolution (R = −0.13, p = 0.038). However, time-to-event analysis did not highlight significant associations with patient outcome in either our internal cohort or TCGA-BRCA dataset. **Conclusions:** Our study suggests a potential role of miR-27a-5p as tumor suppressor miRNA in breast cancer. Further investigations may help define its biomarker potential in each breast cancer subtype, and identify other molecular partners as targets for new interventions.

## 1. Introduction

Breast cancer (BC) is the most common malignancy among women globally and continues to be the primary cause of cancer-related deaths, largely due to the onset of metastases [1,2]. Compared to other solid tumors, where metastatic recurrence may happen earlier, breast cancer exhibits a broad range of relapse times, spanning from months to decades following surgery and treatment of the primary tumor [3,4,5,6]. The underlying reasons for this unique recurrence pattern remain unclear, but they are likely associated with tumor heterogeneity and molecular differences among the different subgroups [5]. Indeed, pathological assessment of the estrogen (ER) and progesterone (PR) receptor status, and the expression and/or amplification of the human epidermal growth factor receptor type 2 (HER2), allows classification into three clinical entities: hormone-receptor (HR)-positive (HR+; ER+, PR+/− and HER2−), HER2-positive (HER2+), and triple-negative (TNBC; ER−, PR− and HER2−) [6,7]. Furthermore, the identification of five intrinsic molecular subgroups of breast cancer based on the PAM50 gene expression classifier—luminal A, luminal B, HER2-enriched, basal-like, and normal-like—has shed light on the intricate molecular heterogeneity within the disease, suggesting that such a diversity may be crucial in determining the prognosis and treatment response of patients [8]. For instance, patients diagnosed with basal-like and HER2-enriched breast cancers tend to experience early relapses within the first 5 years following diagnosis [6,9,10,11]. These subtypes are associated with a more aggressive behavior of the disease and may require more intensive treatment strategies [12]. On the other hand, patients with luminal cancers, particularly the luminal A subtype, generally exhibit a more favorable prognosis. Luminal cancers are typically hormone receptor-positive and respond well to hormone-based therapies, which have contributed to improved outcomes for these patients. However, up to 30% of HR+ tumors eventually develop mechanisms of resistance to endocrine therapy, which may ultimately lead to a deadly metastatic disease. Thus, since breast cancer remains the most frequently diagnosed cancer in females, identifying the drivers and early predictors of recurrence in these patients is of paramount importance.

MicroRNAs (miRNAs) are short (18–25 nucleotides) highly conserved non-coding RNAs that fine-tune gene expression post-transcriptionally through complementary binding with the 3′-untranslated region (3′UTR) of downstream target mRNAs [13]. It has been widely proven that global miRNA deregulation is a typical trait of human malignancies and may strongly affect each functional cell capability encompassing differentiation, proliferation, apoptosis, migration, invasion, and metastasis, as well as lead to chemotherapy resistance [14]. More importantly, an extensive literature has demonstrated that miRNAs may serve as promising biomarkers to refine diagnosis, prognosis, and prediction of treatment efficacy in BC [15,16] and other cancer types.

In our previous work, we derived the global miRNA expression profile of a panel of breast cancer cell lines, each representing a different molecular subtype, and of correspondent, in vitro-derived mammospheres, likely enriched in BC stem cells, with the aim to identify as yet functionally uncharacterized microRNAs in human breast carcinogenesis and evaluate their clinical relevance [17].

Among the most differentially expressed miRNAs, miR-27a-5p showed a significant downregulation in all mammospheres compared to correspondent parental cells. Moreover, such a significant downregulation was also observed in tumor samples compared to normal breast tissues in the Cancer Genome Atlas Breast Invasive Carcinoma (TCGA-BRCA) dataset [18].

In this scenario, we investigated the putative involvement of miR-27a-5p in promoting a migration-prone phenotype of breast cancer cells and sought to determine whether miR-27a-5p expression levels are associated with tumor clinicopathological characteristics.

## 2. Material and Methods

Breast cancer cell lines. Commercially available cell lines consisting of triple negative breast cancer cell lines (TNBC) SUM159 and MDA-MB-231 were grown according to recommended culture conditions (ATCC, American Type Culture Collection).

miR-27a-5p inhibitor in vitro delivery. To achieve the transient modulation of endogenous miR-27a-5p levels, 50 nM miRCURY LNA miR-27a-5p inhibitor (GeneGlobe ID—YI04103216, Qiagen, Hilden, Germany), and correspondent either 50 nM miRCURY LNA miRNA Inhibitor Control A (GeneGlobe ID—YI00199006, Qiagen, Hilden, Germany) or 50 nM AllStar Cell Death siRNA (GeneGlobe ID—SI04939025, Qiagen, Hilden, Germany) (as transfection control) negative controls were delivered by using HiPerFect Transfection Reagent (Qiagen, Hilden, Germany), according to the manufacturer’s protocol (Qiagen, Hilden, Germany). Transfection efficiency was ascertained by RT-qPCR at different time intervals. For each cell line, miR-27a-5p levels in inhibitor-transfected cells were calculated by a relative quantification method as fold change (2^−ΔΔCT^) to correspondent negative control c(-)-transfected cells [17,19,20].

Cell growth assay. Upon 24/48 h of transfection (depending on knockdown efficiency as measured by RT-qPCR) in 96-well plates, WST-1 proliferation reagent (Roche Diagnostics, Mannheim, Germany) was added to the wells every 24 h, up to 96 h according to the manufacturer’s recommendations (Roche Diagnostics, Mannheim, Germany). Absorbance was measured at a wavelength of 450 nm with a reference wavelength at 620 nm. Differences between the miR-27a-5p knockdown and inhibitor control cells were compared by one-way ANOVA (n = 6).

Wound-healing assay. Upon 24/48 h of transfection (depending on knockdown efficiency as measured by RT-qPCR) in 12-well plates, cell migration was assessed by wound-healing assay at different time intervals after introducing scratches [21,22,23,24]. The area of the scratches was determined using the Image J (v. 1.52t) plugin “MRI Wound Healing Tool”, and for each time point, the remaining area relative to the 0 h time point was calculated. Differences between knockdown and control cells were compared by means of one-way ANOVA (*** *p* < 0.0001; n = 4).

CSS_cohort: Our study population included 243 patients grouped as follows: 223 invasive breast cancer cases without metastases at diagnosis (M0), of whom 45 developed metastases during the whole follow-up (M0 > M1, 19%; Incidence Rate per 100 PY = 3.25), 9 invasive breast cancers with synchronous metastases (M1), and 11 non-invasive breast lesions (PreBr). As controls, 13 normal breast tissues (NBTs) from reductive mammoplasties from healthy individuals not suffering from any breast pathology were also profiled. Due to legal reasons, only women older than 18 years of age and with tumors greater than 1.0 cm in diameter could be included in this study.

Clinicopathological data were reported in accordance with recommendations for tumor biomarker prognostic studies [25]: positive hormone receptor BCs were cases showing estrogen receptor (ER)-expressing neoplastic cells ≥ 1% as per international guidelines [26]; HER2 status assessment was performed according to standard recommendations [27]. Tumors were staged according to the 7th version staging system of the World Health Organization (WHO) [28]. All patients underwent adequate local treatment (breast conserving surgery or total mastectomy) plus sentinel node biopsy or complete axillary dissection. Post-surgery treatments were administered according to national and international guidelines. Progression was defined as evidence of loco-regional recurrence and/or distant metastases over 4 months from diagnosis and after curative-intent surgical treatment.

All investigations were carried out in accordance with international (Helsinki Declaration 7th rev, 2013, EU Directive 2004/23/EC) and Italian (D. Lgs. 30/06/2003, n. 196) regulations for research on human subjects. All experimental protocols were reviewed and approved by the Ethical Committee of the Fondazione IRCCS “Casa Sollievo della Sofferenza” (Protocol code: MO-PR-8). Prior written informed consent was obtained from all patients and healthy individuals in compliance with the experimental protocol as approved by the Ethical Committee.

Human tissue collection. The breast tissues included in this study were collected at the Breast Unit, Fondazione IRCCS Casa Sollievo della Sofferenza, from January 2006 to December 2014. After pathological evaluation, tissue samples were snap-frozen in liquid nitrogen and stored at −80 °C until used. For each sample, a 5 μm hematoxylin/eosin-stained section was checked by light microscopy to select areas with a viable cancer cell content of at least 70% for tumors, and to confirm the absence of anomalies in the normal specimens, obtained from reductive mammoplasties (i.e., normal breast tissues, NBTs).

Analysis of miR-27a-5-p expression by RT-qPCR. Total RNA from tissues and cells was isolated by using TRIzol reagent (Thermo Fisher Scientific, Waltham, MA, USA) according to the manufacturer’s instructions. Tissue RNA quality was evaluated by using the 2100 Expert Analyzer (Agilent Technology, Santa Clara, CA, USA), and only RNAs with an RNA Integrity Number (RIN) ≥ 7.0 were used for the expression analyses. RNA concentration was quantified by the absorbance measurement at 260 and 280 nm using the NanoDropTM.1000 spectrophotometer (hermo Fisher Scientific, Waltham, MA, USA). A quantification method with standard curves was applied to measure expression levels of miR-27a-5p as previously described [20,29]. Briefly, 10 ng of total RNA were reverse transcribed to single-stranded cDNA using TaqMan MicroRNA Reverse Transcription Kit (Thermo Fisher Scientific, Waltham, MA, USA) and 5× specific stem-loop RT primers for both miR-27a-5p and the RNU48 endogenous control, according to the manufacturer’s instructions (Thermo Fisher Scientific, Waltham, MA, USA). miR-27a-5p was estimated using TaqMan MicroRNA Assays (assay ID: 002445) and normalized to an RNU48 endogenous control (assay ID: 001006; Thermo Fisher Scientific, Waltham, MA, USA). For both miR-27a-5p and the RNU48 endogenous control, standard curves were built by plotting the threshold cycle (Ct) values against log10 of the copy number and fitting by linear least squares regression. For each breast tumor and NBT sample, miR-27a-5p levels were determined as the ratio of the miR-27a-5p copy number to the RNU48 copy number, multiplied by 1000 for more straightforward tabulation (i.e., miR-27a-5p/RNU48) × 1000) [20,29].

To test transfection efficiency, 1 μg of total RNA was reverse transcribed using miScript II RT Kit (Qiagen, Hilden, Germany) according to the manufacturer’s protocol. qPCR analyses were carried out using miScript Primer Assays (Qiagen, Hilden, Germany) and the miScript SYBR Green PCR Kit (Qiagen, Hilden, Germany) on a LightCycler 480 Real-Time PCR System (Roche Diagnostics, Mannheim, Germany) according to the manufacturer’s protocol. For each cell line, relative miR-27a-5p expression levels in transfected cells were calculated using the 2−ΔΔCT method according to [17,19,20], as follows: miR-27a-5p Ct values were first normalized to SNORD61 and SNORD95 housekeeping genes, and then the fold change between miR-27a-5p-treated cells and correspondent negative controls was calculated.

Selection of the Cancer Genome Atlas Breast Invasive Carcinoma (TCGA-BRCA) cohort. We downloaded miRNA data and publicly available clinical information from the TCGA data portal to build an external validation cohort. The log2 read counts were used for miRNA expression analysis. Of the 1053 women with breast cancer, not treated with neoadjuvant therapy, 877 were classified without metastases (AJCC Pathologic staging M0) and had complete follow-up data. Based on miR-27a-5p expression data availability, a cohort of 561 patients was ultimately selected for subsequent analyses. Characteristics of this cohort are reported in Supplemental Table S3).

Statistical Analysis: Patient baseline characteristics were reported as median along with interquartile range (IQR, i.e., first–third quartiles) or frequencies and percentages for continuous and categorical variables, respectively. Comparisons between miR-27a-5p levels and patient clinicopathological information were assessed by Pearson correlation coefficient and two-sample *t*-test (or ANOVA model as appropriate) for continuous and categorical variables, respectively. The assumption of normal distribution was checked by means of Q–Q plots and Shapiro–Wilks test. Time-to-event analysis was performed in patients without metastases at diagnosis (N = 223) by univariable proportional hazards Cox regression models. Risks were reported as hazard ratios (HR) along with their 95% confidence intervals (95%CI). Overall survival (OS) was defined as the time between the enrollment date and cancer-related death. Progression-free survival (PFS) was defined as the time between the enrollment date and the tumor progression. Metastasis-free survival (MFS) was defined as the time between the enrollment date and the development of distant metastases. All statistical analyses were performed using SAS Release 9.4 (SAS Institute, Cary, NC, USA).

## 3. Results

**Figure 1:**
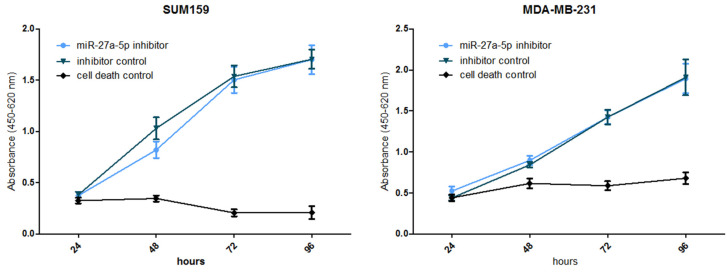
Effect of miR-27a-5p knockdown on cell proliferation in two triple negative breast cancer cell lines. Differences between the miR-27a-5p knockdown and inhibitor control cells were compared by one-way ANOVA (n = 6).

**Figure 2:**
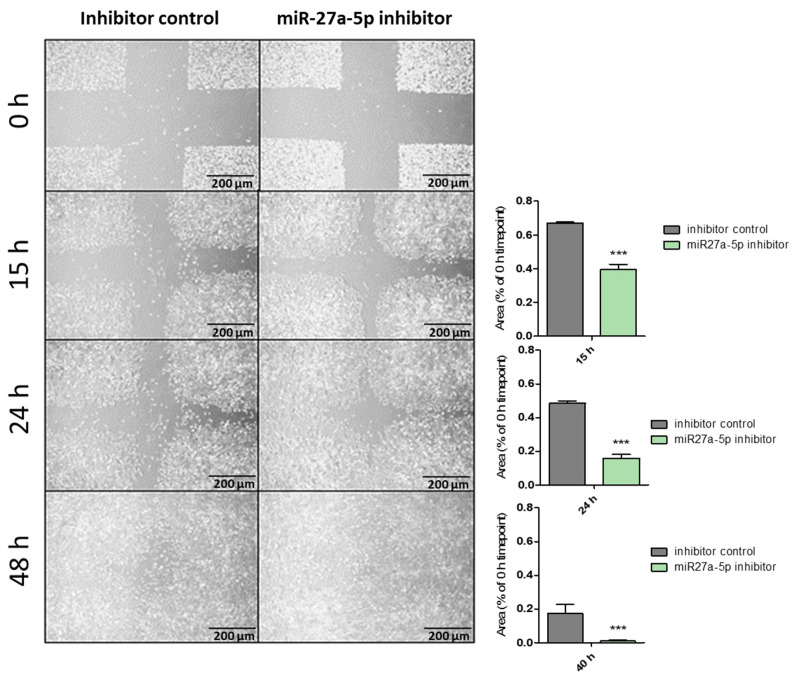
Effect of miR-27a-5p knockdown on cell migration of the triple negative breast cancer cell line SUM159. The area of the scratches was determined using the Image J plugin “MRI Wound Healing Tool” and, for each time point, the remaining area relative to the 0 h (0 h) time point was calculated. Differences between knockdown and control cells were compared by means of one-way ANOVA (*** *p* < 0.0001; n = 4).

**Figure 3:**
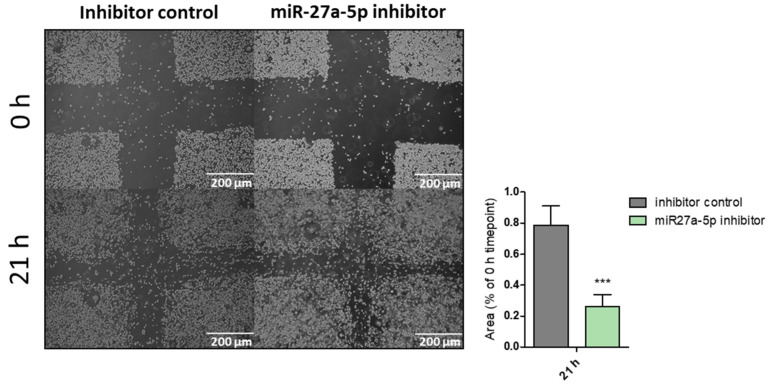
Effect of miR-27a-5p knockdown on cell migration of the triple negative breast cancer cell line MDA-MB-231. The area of the scratches was determined using the Image J plugin “MRI Wound Healing Tool” and, for each time point, the remaining area relative to the 0 h time point was calculated. Differences between inhibited and control cells were compared by means of one-way ANOVA (*** *p* ≤ 0.001; n = 4).

Effects of miR-27a-5p in Triple Negative Breast Cancer (TNBC) cell lines. According to our preliminary data, miR-27a-5p showed downregulation in all cancer stem-cell-enriched mammospheres as compared to parental adherent cells. Since deregulation of stemness pathways seems to be correlated to metastases onset and poor overall survival, we made use of two different triple negative breast cancer (TNBC) cell lines TNBC), consisting of SUM159 and MDA-MB-231, carrying low endogenous expression levels of miR-27a-5p (Supplemental Figure S1), and evaluated the effects on cell phenotypes in terms of enhanced cell proliferation, and migration upon transient inhibition of miR-27a-5p. Cell proliferation was measured by a WST-1 assay (Roche Diagnostics, Mannheim, Germany) after 96 h of transfection (Figure 1). Apparently, miR-27a-5p inhibition did not cause a significant growth difference compared to control cells. We moved forward and evaluated the ability of miR-27a-5p to affect the motility of cells lines, thereby promoting a metastatic phenotype. To this aim, SUM159 and MDA-MB-231 cell lines were tested by scratch assays after reaching miR-27a-5p inhibition. As expected, both cell lines (Figure 2 and Figure 3) showed a significant increase in their migratory ability following miR-27a-5p suppression, thus suggesting a tumor suppressive role of miR-27a-5p.

miR-27a-5p is downregulated in tumors with metastatic potential. The expression analysis of miR-27a-5p (hsa-miR-27-5p/RNU48x1000) was carried out in 232 breast cancers (M0, M0 > M1 and M1) of the CSS cohort, in 11 non-invasive breast lesions and in the normal breast tissues obtained from 13 reductive mammoplasties (i.e., glandular tissue, NBTs). Descriptive characteristics of the CSS cohort are reported in Supplemental Table S1. Considering the whole cohort, it emerged that miR-27a-5p exhibited higher levels in normal breast tissues (NBTs) (Median 2.28, IQR 1.50–5.40) and pre-invasive breast lesions (Median 3.32, IQR 1.68–4.32) in comparison with tumor samples. In particular, miR-27a-5p expression was diminished in patients with synchronous (M1, Median 1.03 IQR 0.83–1.58) or metachronous (M0 > M1, Median 1.83, IQR 1.29–3.17) metastases than in patients free from metastases after a 5-year follow-up (M0, Median 2.17, IQR 1.19–3.64) (Table 1). This suggests that miR-27a-5p expression (log values) is negatively correlated with breast pathology evolution (R = −0.13, *p* = 0.038).

Association between miR-27a-5p and patient clinicopathological features. Next, we investigated the correlation between miR-27a-5p expression and tumor clinicopathological characteristics. Consistent with the above results, miR-27a-5p levels showed a significant correlation with lymph node status classification (*p* = 0.003), being more expressed in N0 (n = 94, median 1.93, IQR 1.19–3.33) and N1 (n = 77, median 2.68, IQR 1.48–4.31) than in N2 (n = 26, median 1.40 IQR 1.02–2.37) and N3 (n = 35, median 1.56 IQR 0.83–3.17) groups, and with Ki67 (R = 0.166, *p* = 0.015), thus supporting the association of its downregulation with a more aggressive behavior of cancer (Table 2).

Evaluation of miR-27a-5p prognostic value in breast cancer cases. The association with time-to-event outcomes was evaluated in the group of patients without metastases at diagnosis (N = 223). However, complete information about overall survival (OS), progression-free survival (PFS) and metastasis-free survival (MFS) for the univariable analysis as well as tumor stage, grading, and Ki67, which were used in multivariable analyses as covariates, were available for 222 and 199 patients, respectively (Supplemental Table S2). Unfortunately, no statistically significant associations with clinical outcome were found for miR-27a-5p. Then, we extended our analysis to the TCGA-BRCA dataset by selecting 561 cases without synchronous metastases, for whom miR-27a-5p expression levels and clinical and follow-up data were available (Supplemental Table S3). In line with the results we obtained in our CSS cohort, no association with survival could be found in the TCGA-BRCA overall population as well as in the molecular subgroups identified by PAM50 (Supplemental Table S4).

## 4. Discussion

Despite advances in our understanding of breast cancer heterogeneity and the mechanisms underlying breast cancer metastases, the accurate prognostication of high-risk individuals is hampered by the lack of robust and reliable biomarkers that may help predict early the risk of metastatic relapse, which may sometimes occur after a prolonged period of undetectable disease following surgery or systemic therapy. MicroRNAs (miRNAs) are capable of simultaneously regulating multiple biological targets, thus influencing numerous cellular and extracellular signaling pathways, the altered expression of which can promote cancer onset and progression. For this reason, miRNAs might represent promising biomarkers for diagnosis, prognosis, and treatment efficacy prediction in both breast cancer and other types of cancer.

To date, only a few reports have reported about deregulation of miR-27a-5p, the passenger strand of pre-miR-27a, in different cancers such as gastric [30], prostate [31], small cell lung cancer [32], head and neck squamous cell carcinoma [33], and gliomas [34]. Indeed, most of the past literature rather focused on the functional significance of the usually dominant guide strand miR-27a-3p, whose upregulation and tumor-promoting role showed relevance in many other tumors, including breast cancer [35]. However, based on our findings, the miR-27a-5p passenger strand may be functionally relevant in breast cancer as well. Moreover, since its downregulation specifically occurs in breast cancer stem cells, whose enrichment has been shown to be more pronounced in TNBC than other subtypes, and negatively correlated with chemotherapy response, disease-free, metastasis-free, and overall survival, miR-27a-5p holds promise as a novel prognostic biomarker in this BC subtype.

In this study, we tested in vitro the ability of miR-27a-5p to promote the development of a migratory phenotype prone to dissemination and metastasis formation in the subtype of triple-negative breast cancer. Following ectopic inhibition of miR-27a-5p in SUM159 and MDA-Mb-231 cell lines, no effects were observed on cell proliferation. Instead, an increased cellular motility upon miR-27a-5p reduction did occur, supporting a specific involvement of miR-27a-5p in the mechanisms underlying cell migration and invasion, and its role as a tumor-suppressive miRNA. In particular, this might suggest that miR-27a-5p functionality in breast cancer might be similar to that observed in gastric and prostate cancer cells [30,31]. For instance, in gastric cancer cells, the suppression of miR-27a-5p appeared to promote both proliferation and cell motility, likely due to the fact that inhibition of miR-27a-5p impaired the suppression of one of its targets, APEX1, which is known to promote epithelial–mesenchymal transition (EMT) and related cell phenotypic changes [30]. Moreover, in prostate cancer, the observed downregulation of miR-27a-5p, due to promoter methylation and c-*MYC* aberrant activation [31], has been proposed as a mechanism that may lead to increased levels of TOP2A, MELK, and CENPF, which represent predicted miR-27a-5p targets associated to poor prognosis [36,37] and development of metastases [38]. Of course, subsequent studies may be aimed at identifying any target mRNA and molecular partners/axis through which miR-27a-5p exerts its regulation in the specific context of breast cancer.

Next, in order to evaluate the association of miR-27a-5p with tumor clinicopathological characteristics, we conducted an expression analysis of miR-27a-5p in a cohort of breast cancer patients including both metastatic (M1, M0 > M1) and non-metastatic (M0) cases. This analysis first confirmed lower levels of miR-27a-5p in carcinomas compared to normal tissues and pre-invasive lesions, supporting its role as a tumor-suppressive miRNA. In addition, miR-27a-5p levels showed a significant inverse correlation with lymph node status classification (*p* = 0.003) and were significantly reduced in patients with synchronous metastases (M1) or who had developed metastases within 5 years of follow-up (M0 > M1) compared to patients who had not in the same time interval. These data confirm the potential involvement of miR-27a-5p in processes underlying the malignant evolution of the disease and the development of metastases.

Despite the lack of statistical significance, our results show that miR-27a-5p expression levels tend to be decreased in metastatic breast cancer as compared with non-metastatic tumors. A potential association with more aggressive breast cancer phenotypes is further supported by miR-27a-5p association with lymph node status and Ki67. As such, although our results seem not to encourage the use of miR-27a-5p as an independent biomarker, we cannot exclude that a prognostic role might be held within a single breast cancer molecular subgroup. To test this hypothesis, we made use of the TCGA-BRCA dataset and the PAM50-based molecular classification of BC cases. We did not find any association between miR-27a-5p and survival across the molecular Basal, HER2amp, LUMA, and LUMB subtypes. However, this might be due to the partial information about overall survival or the absence of data availability on progression for the whole TCGA-BRCA dataset. Thus, miR-27a-5p biomarker potentials need to be further investigated in studies specifically designed to evaluate its expression in each of the breast cancer subtypes.

In conclusion, both in vitro and patient studies highlight the biological role of miR-27a-5p in breast pathology; consequently, by focusing our attention on the signaling pathways governing metastasis formation in which miR-27a-5p is involved may allow us to identify in miR-27a-5p itself and in its potential molecular partners targets for new targeted therapies.

## Figures and Tables

**Table 1:** Assessment of a linear trend of miR-27a-5p expressions across tumor stages overall.

Sample	Group	miR-27a-5p Median [IQR]	Test for Linear Trend (on Log Values)	
			R	*p*-Value
Overall (N = 256)	0- NBT (N = 13)	2.28 [1.50–5.40]	−0.13	0.038
	1- Non-invasive tumors (N = 11)	3.32 [1.68–4.32]		
	2- M0 (N = 178)	2.17 [1.19–3.64]		
	3- M0->M1 (N = 45)	1.83 [1.29–3.17]		
	4- M1: Synchronous metastasis (N = 9)	1.03 [0.83–1.58]		

**Table 2:** Association between miR-27a-5p and clinical–pathological characteristics of all recruited patients.

Clinical Variable	Level	Number of Patients #	Median [IQR] of miR-27a-5p or Correlation Coefficient (R)	*p*-Value *
Age (years)	R correlation coefficient	242	R = −0.02	0.719
Menopause—N (%)	No	72	2.24 (1.26–3.33)	0.594
	Yes	164	1.94 (1.10–3.76)	
TH—N (%)	IDC	212	2.28 (1.20–3.78)	0.101
	ILC	14	1.26 (0.70–1.95)	
	IDC + ILC	5	1.33 (1.23–1.58)	
	Others	11	3.32 (1.68–4.32)	
Site of onset—N (%)	Right	113	1.76 (1.18–3.63)	0.402
	Left	117	2.34 (1.14–3.58)	
	Bilateral	1	2.78 (2.78–2.78)	
Type of Surgery—N (%)	MAST + LND	125	2.65 (1.30–4.31)	0.003
	MAST + SN	28	2.26 (1.63–3.79)	
	QUADR	4	2.18 (1.38–3.30)	
	QUADR + LND	54	1.49 (0.78–2.10)	
	QUADR + SN	20	1.88 (1.16–2.70)	
Tumor size (cm)	R correlation coefficient	231	R = 0.01	0.923
T Classification—N (%)	T1c	61	1.93 (1.14–3.33)	0.995
	T2	124	1.96 (1.16–3.39)	
	T3	13	1.82 (1.33–3.68)	
	T4	33	2.32 (1.30–4.31)	
N classification—N (%)	N0	94	1.93 (1.19–3.33)	0.003
	N1	77	2.68 (1.48–4.31)	
	N2	26	1.40 (1.02–2.37)	
	N3	35	1.56 (0.83–3.17)	
Lymph node status—N (%)	Negative	94	1.93 (1.19–3.33)	0.944
	Positive	148	2.14 (1.11–3.78)	
M classification—N (%)	M0	222	2.11 (1.21–3.61)	0.240
	M1	9	1.03 (0.83–1.58)	
Tumor stage—N (%)	1	35	1.77 (0.85–3.14)	0.028
	2A	82	2.28 (1.23–3.25)	
	2B	37	2.61 (1.55–3.95)	
	3A	15	1.64 (1.08–2.37)	
	3B	23	3.58 (1.49–5.19)	
	3C	32	1.65 (0.83–3.17)	
	4	7	1.03 (0.83–1.58)	
Tumor grading—N (%)	1	23	2.41 (1.16–3.81)	0.618
	2	119	1.96 (1.22–3.64)	
	3	89	1.83 (1.14–3.60)	
ERc—N (%)	Negative	53	1.91 (1.14–3.95)	0.637
	Positive	178	1.96 (1.19–3.58)	
PgR—N (%)	Negative	71	1.83 (1.18–3.43)	0.896
	Positive	160	2.15 (1.12–3.64)	
HER2—N (%)	AMP	54	2.02 (1.16–3.51)	0.990
	NEG	165	1.94 (1.17–3.60)	
Ki67	R correlation coefficient	216	R = 0.166	0.015
OT—N (%)	No	54	1.86 (1.14–3.07)	0.988
	Yes	159	2.12 (1.21–3.61)	
CT-ADJ—N (%)	No	40	2.36 (1.73–3.40)	0.421
	Yes	171	1.82 (1.11–3.60)	
RT—N (%)	No	55	2.44 (1.19–3.43)	0.660
	Yes	152	1.80 (1.16–3.59)	

Abbreviations—IQR: Interquartile range (i.e., first–third quartiles). # number of patients for whom clinical variable information was available. * *p*-values from ANOVA model and Pearson correlation coefficient on miR-27a-5p log values for continuous and categorical clinical variables, respectively.

## Data Availability

Preliminary data for this study have been generated in this published article: “Schwarzenbacher D, Klec C, Pasculli B, Cerk S, Rinner B, Karbiener M, Ivan C, et al. MiR-1287-5p inhibits triple negative breast cancer growth by interaction with phosphoinositide 3-kinase CB, thereby sensitizing cells for PI3Kinase inhibitors. Breast Cancer Res. 2019; 21(1):20. doi: 10.1186/s13058-019-1104-5” [17].

## Supplementary Materials

The following supporting information can be downloaded at: https://www.mdpi.com/article/10.3390/biomedicines12112625/s1, Figure S1: Endogenous expression of miR-27a-5p in breast cancer cell lines; Table S1: Baseline clinical-pathological characteristics; Table S2: Survival Analyses in the CSS_cohort; Table S3: Baseline clinical-pathological characteristics in TCGA-BRCA dataset; Table S4: Survival Analyses in TCGA-BRCA cohort.
