# Supplementary figures and images for: Tumor Suppressor miR-27a-5p and Its Significance for Breast Cancer
Source: Biomedicines. 2024 Nov 17;12(11):2625. doi: 10.3390/biomedicines12112625 (PMC11592003; doi:10.3390/biomedicines12112625)

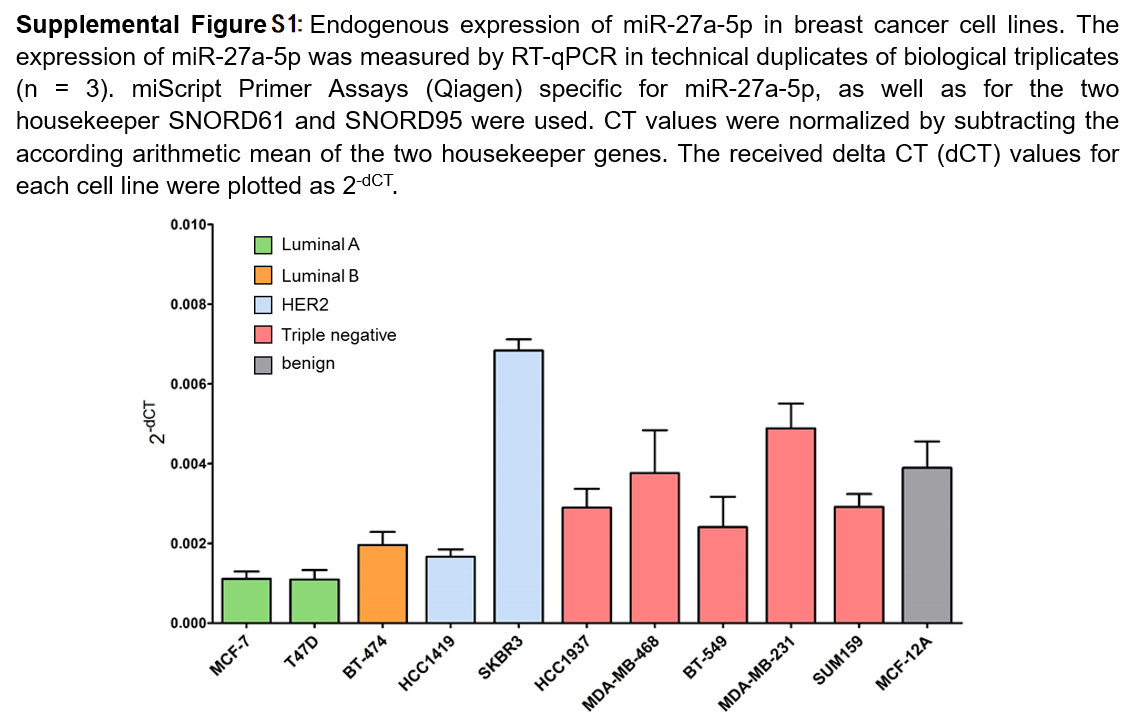

Supplement: Supplementary file 1 [file biomedicines-12-02625-s001.zip › Supplemental_Figure S1.png]
